# Bilateral Multiple Chalazia After Adalimumab Therapy for Uveitis Associated With Generalized Pustular Psoriasis: A Case Report

**DOI:** 10.7759/cureus.86646

**Published:** 2025-06-24

**Authors:** Azusa Yamagishi, Tomomi Kaiho, Jiro Yotsukura, Jun-ichiro Ikeda, Takayuki Baba

**Affiliations:** 1 Department of Ophthalmology and Visual Science, Chiba University Graduate School of Medicine, Chiba, JPN; 2 Department of Diagnostic Pathology, Chiba University Graduate School of Medicine, Chiba, JPN

**Keywords:** adalimumab (humira), certolizumab pegol, chalazion, generalized pustular psoriasis (gpp), glaucoma, reactive granulomatous dermatitis, secondary failure, steroid-induced glaucoma, tnf-inhibitor, uveitis

## Abstract

Adalimumab is a widely used tumor necrosis factor (TNF) inhibitor that is rarely associated with ophthalmic adverse events. Herein, we report a case of multiple bilateral chalazia that developed after adalimumab therapy. A 32-year-old woman was treated with adalimumab for uveitis associated with generalized pustular psoriasis, with coexisting psoriatic arthritis. She initially experienced systemic and ocular symptom improvement after starting adalimumab; however, five months later, bilateral chalazia resistant to conservative management developed, requiring surgical intervention. Excisional surgery was performed, and a histopathological examination revealed granulomatous inflammation with epithelioid cells, multinucleated giant cells, plasma cells, and lymphocytes consistent with chalazia. Persistent blepharitis and residual chalazia were observed postoperatively. A subsequent relapse of uveitis, skin lesions, and joint symptoms indicated secondary failure of adalimumab; therefore, treatment was switched to certolizumab pegol. Thereafter, both systemic and ocular symptoms markedly improved. Because of the unusual size, bilaterality, multiplicity, and clinical course of the disease, immune imbalance induced by adalimumab may have contributed to chalazia development.

## Introduction

Adalimumab, a tumor necrosis factor (TNF) inhibitor, is a biologic agent used to treat various immune-mediated diseases. In ophthalmology, adalimumab is indicated for the treatment of noninfectious uveitis when corticosteroids and conventional immunosuppressants are insufficient [1]. Ocular adverse events associated with adalimumab are rare, and only limited reports are available [2].

We encountered a patient with refractory uveitis associated with generalized pustular psoriasis (GPP), who initially responded well to adalimumab but subsequently developed unusually large, bilateral, and multiple chalazia that required surgical excision. Chalazion excision is typically associated with favorable outcomes. Contrary to previously reported cases with favorable postoperative outcomes, this case showed persistent blepharitis and residual chalazia following surgery [3,4]. Eventually, the patient experienced worsening uveitis and systemic symptoms, which led to the diagnosis of secondary failure of adalimumab. Following a switch to certolizumab pegol, not only the uveitis and GPP symptoms but also the chalazia improved.

Large, bilateral, multiple, and refractory chalazia have been associated with underlying local or systemic immune abnormalities in previous reports [5-7]. Given the distinctive clinical course and lesion characteristics, a possible association between adalimumab and chalazion development was considered, and this case is therefore reported.

## Case presentation

A 32-year-old woman had a history of psoriasis vulgaris diagnosed at age 20 years and uveitis diagnosed at age 26 years; however, she discontinued ophthalmologic follow-up 1 year after the diagnosis of uveitis. At age 30, she resumed treatment for uveitis; however, because of steroid-induced glaucoma resulting from prolonged topical steroid use, she was referred to our department. She had undergone phacoemulsification for steroid-induced cataracts in both eyes at another hospital. She had no history of allergies, smoking, or systemic diseases other than psoriasis vulgaris. In addition to the topical agents prescribed by the dermatology clinic, she independently purchased a generic version of apremilast (Glenmark Pharma, Mumbai, India) from an online website for psoriasis treatment.

Because comprehensive systemic management was deemed necessary, we consulted with a rheumatologist and a dermatologist. Physicians in the rheumatology department diagnosed psoriatic arthritis based on joint ultrasound examination, and those in the dermatology department suspected GPP because of the presence of generalized erythema with pustules. However, a definitive diagnosis of GPP could not be made initially because the patient refused to undergo a skin biopsy.

**Figure 1:**
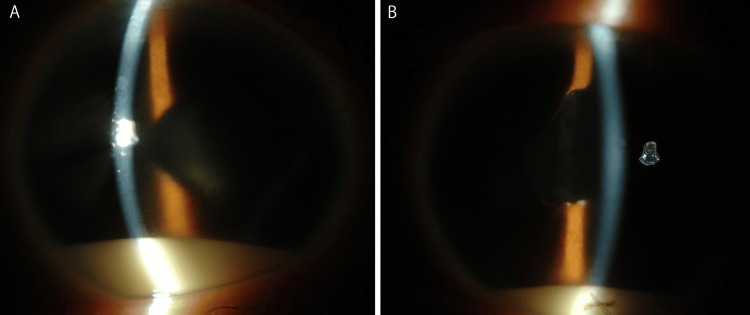
Hypopyon during an inflammatory attack in both eyes A: Right eye. B: Left eye. Both eyes have hypopyons and fluid formation. Subconjunctival dexamethasone injections were administered bilaterally to improve inflammation; however, IOP elevation persisted.

During the first ophthalmologic examination, the best-corrected visual acuity values were 0.8 in the right eye and 1.0 in the left eye. Intraocular pressure (IOP) values were 28 mmHg in the right eye and 38 mmHg in the left eye despite the use of antiglaucoma eye drops and oral carbonic anhydrase inhibitors. When the topical steroid was changed from 0.1% betamethasone to 0.01% betamethasone, IOP decreased; however, an inflammatory attack with hypopyon occurred (Figure 1). During follow-up, simultaneous control of IOP and inflammation was difficult.

At 3 months after the initial visit, despite discontinuation of topical steroids, IOP in the left eye was more than 30 mmHg. Surgery for glaucoma in the left eye and anti-inflammatory treatment other than steroids were deemed necessary. Although cyclosporine was initiated, it was discontinued because of its side effects. Additionally, because of financial constraints and work-related issues, the patient initially declined biologic therapy and surgery.

However, the patient eventually consented to surgery because of progressive visual field loss. In the left eye, trabeculectomy was performed three months after the IOP increase, and in the right eye, microhook trabeculectomy was performed one month after the IOP increase. Both procedures resulted in good IOP control. Postoperatively, the patient used antibiotics and steroid eye drops for both eyes.

Eventually, a skin biopsy was performed, and the results confirmed GPP. Therefore, the patient was eligible for public medical assistance, and adalimumab therapy was initiated one week after trabeculotomy. Topical 0.1% betamethasone was gradually tapered thereafter, and uveitis did not recur. Skin lesions and joint pain also improved after adalimumab initiation.

Five months after starting adalimumab, the patient developed chalazia of both eyelids. The right eyelid presented with two lesions measuring 12 mm × 8mm in total, while the left eyelid had three lesions measuring 18 mm × 15 mm. Despite warm compresses, eyelid hygiene, and treatment comprising 0.1% fluorometholone drops and neomycin sulfate/methylprednisolone ointment, the chalazia did not improve. Therefore, surgical excision was performed five months after the onset.

During excision, fatty components were observed within the lesions. Direct measurement of the excised capsule of the largest left lesion indicated a length of 8 mm, and the histopathological examination indicated a specimen size of 8 mm × 10 mm. The right lesion was not measured during surgery or the histopathological analysis. The histopathological examination revealed granulomatous inflammation characterized by infiltration of epithelioid cells, multinucleated giant cells, plasma cells, and lymphocytes. The histological examination results were consistent with chalazia.

**Figure 2:**
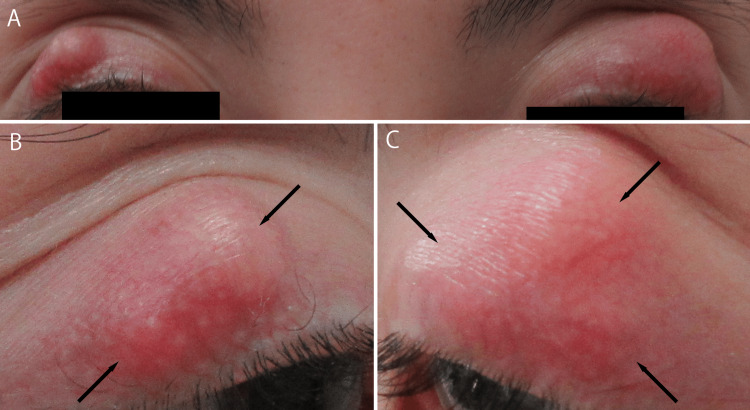
Chalazia of the upper eyelids A: Overall view. B: Right upper eyelid. C: Left upper eyelid. Red and swollen lesions are observed bilaterally. Two chalazia on the right upper eyelid and three on the left upper eyelid form contiguous masses. The right-sided mass measured 12 mm × 8 mm, and the left-sided mass measured 18 mm × 15 mm. The arrows indicate individual lesions.

**Figure 3:**
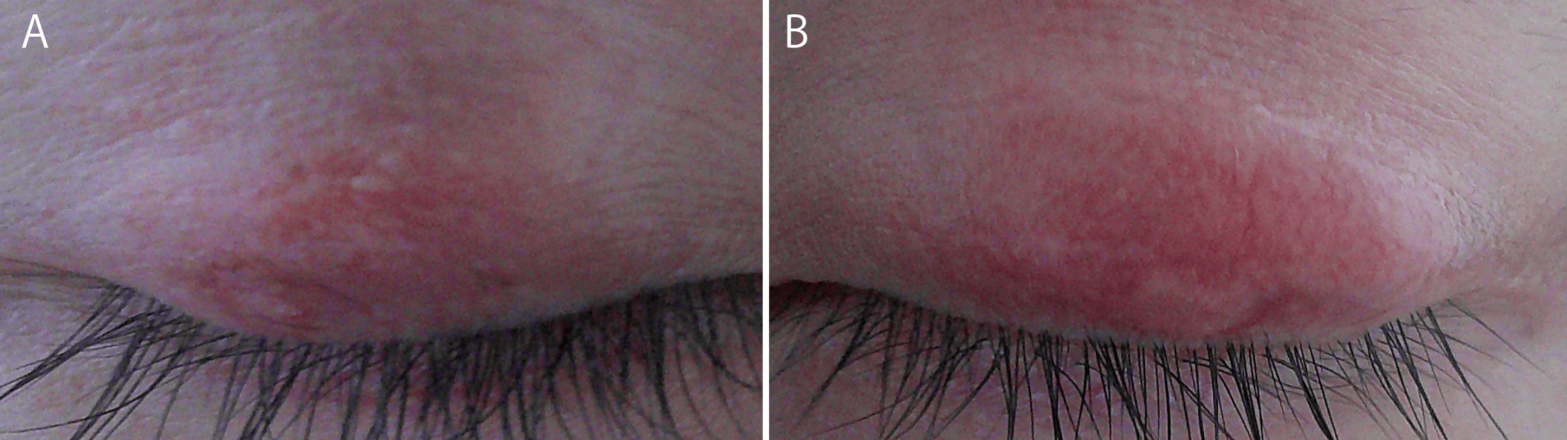
Upper eyelids during the early postoperative period A: Right upper eyelid (2 weeks postoperatively). B: Left upper eyelid (4 weeks postoperatively). Although the postoperative time points differed slightly, both images show early postoperative findings. Redness persists in both eyes, and swelling is more pronounced on the left upper eyelid.

**Figure 4:**
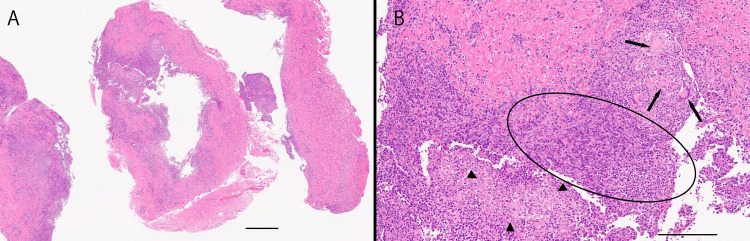
Histopathological findings of the right chalazion A: Low magnification (Hematoxylin-Eosin stain). The specimen exhibits a capsule-like structure with a central cavity. Fibrous tissue, eosinophilic on H&E staining, is infiltrated by basophilic inflammatory cells (scale bar = 500 µm). B: High magnification (Hematoxylin-Eosin stain). Granulomatous inflammation with epithelioid cells, multinucleated giant cells, plasma cells, and lymphocytes is observed infiltrating eosinophilic fibrous tissue and skeletal muscle. Basophilic lymphocytes and plasma cells (oval) represent the predominant inflammatory components, with multinucleated giant cells (arrows) and epithelioid cells (triangles) supporting the diagnosis of a chalazion (scale bar = 200 µm).

During surgery of the left eye, the largest chalazion was excised; however, multiple lesions were subsequently identified. The two smaller chalazia were not excised, and significant blepharitis persisted postoperatively (Figures 2-4).

Despite suspicion that the chalazia development was a potential adverse effect of adalimumab, uveitis remained well-controlled without recurrence. Therefore, the relationship between chalazia and adalimumab was unclear, and adalimumab was continued at that time.

**Figure 5:**
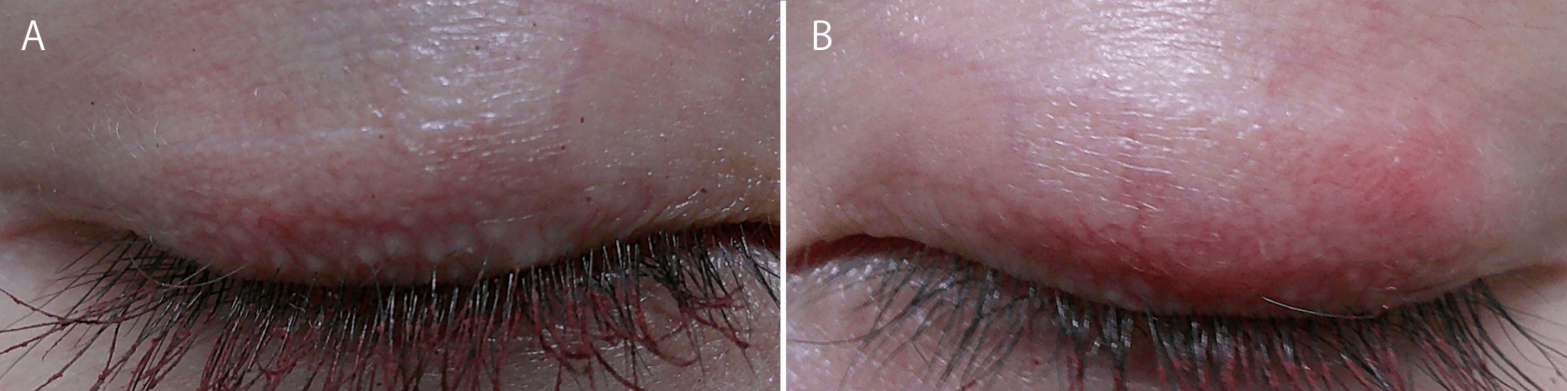
Upper eyelids, six months after surgery A: Right upper eyelid. B: Left upper eyelid. At approximately 6 months postoperatively, the redness improved in both eyes. The size of the mass on the left upper eyelid decreased. On palpation, a slight mass was detectable at the tarsal plate; however, it was not bothersome to the patient.

However, at two months postoperatively, both uveitis and skin and joint symptoms worsened, leading to the diagnosis of secondary failure of adalimumab. Therefore, treatment was switched to certolizumab pegol by the physicians in the rheumatology department. Eventually, both uveitis and systemic symptoms, as well as residual chalazia and blepharitis, improved (Figure 5).

## Discussion

Chalazion is a common disease characterized by granulomatous inflammation caused by the obstruction of the meibomian glands in the eyelids. However, in severe cases involving bilateral and multiple chalazia, underlying local or systemic immune abnormalities should be considered. A large-scale study conducted in India reported that the prevalence of chalazion was 0.57%, and that only 21.48% and 3.03% of chalazion cases comprised bilateral involvement and multiple lesions, respectively. Therefore, most cases are solitary and unilateral [8]. Local inflammatory conditions, such as blepharitis and rosacea, have been reported as major risk factors for chalazion, and cases of bilateral, recurrent, and multiple chalazia associated with ocular rosacea have been documented [5,9]. Additionally, one case report described the development of large bilateral chalazia after bortezomib therapy and indicated that TNF promotion was a possible contributing factor [6]. Recurrent, multiple, large chalazia have been observed in patients with hyperimmunoglobulin E (IgE) syndrome [7]. These findings suggest that not only local but also systemic immune dysregulation may contribute to chalazion development. Chalazia, which are large, bilateral, multiple, and refractory lesions, may be strongly associated with underlying immune abnormalities.

In the present case, the clinical features and disease course suggested a potential association between adalimumab therapy and chalazion development. A previous study defined chalazia larger than 7 mm as large [10]. In our case, the lesions measured 12 × 8 mm on the right and 18 × 15 mm on the left, indicating a considerably large size. Furthermore, multiple chalazia appeared simultaneously on both eyelids, which is a rare feature [8]. Therefore, surgical excision, which is considered to have a high cure rate, was performed; however, residual lesions and prolonged blepharitis persisted postoperatively [3,4]. These features (large size, bilaterality, multiplicity, and refractoriness) supported the suspicion of underlying immune abnormalities in this case. Chalazia developed during a period when uveitis and psoriasis were well-controlled, and blepharitis was not observed prior to their onset. Therefore, these conditions were considered unlikely to be direct causes. Moreover, the gradual improvement of residual chalazia and blepharitis after switching from adalimumab to certolizumab pegol further supported an involvement of adalimumab in chalazion development. Based on these findings, it was concluded that chalazia in this case may have been related to systemic immune dysregulation induced by adalimumab.

Biologic agents are known to cause inflammatory adverse events because of the overproduction or imbalance of inflammatory cytokines; however, reports that have specifically linked biologic agents to chalazion are lacking [11,12]. Among the inflammatory skin-related adverse events associated with biologic agents, reactive granulomatous dermatitis and paradoxical adverse effects have been reported. Reactive granulomatous dermatitis encompasses a spectrum of conditions, including interstitial granulomatous dermatitis, palisaded neutrophilic granulomatous dermatitis, and interstitial granulomatous drug reactions [12]. In contrast, paradoxical adverse effects are defined as new onset or worsening of immune-mediated diseases in response to the use of the same class of agent for treatment [11]. Chalazia are not included in either of these categories. The histopathological findings of this case were consistent with chalazia, and the clinical findings supported this diagnosis. TNF-induced and interferon-induced cytokine dysregulation attributable to biologic agents may have contributed to chalazion formation in this patient.

In this case, because of the secondary failure of adalimumab, treatment was switched to certolizumab pegol after consultation with physicians in the departments of rheumatology and dermatology. In the field of ophthalmology, infliximab and adalimumab are biologic agents with the strongest evidence of effectiveness for uveitis [13]. However, a wider range of biologic agents, including TNF inhibitors, interleukin (IL)-17 inhibitors, and IL-23 inhibitors, are utilized for GPP [14]. Switching to a different class of biologic agents is generally considered effective when secondary failure occurs [15]. Although switching from adalimumab to the IL-17 inhibitor ixekizumab was initially considered, a within-class switch to another TNF inhibitor, certolizumab pegol, was ultimately chosen because of the strong evidence of TNF inhibitor effectiveness for uveitis. Although certolizumab pegol is not widely used to treat uveitis, several studies have demonstrated its efficacy [16-18]. One report has described the successful use of certolizumab pegol for uveitis associated with GPP [19]. In this case, switching to certolizumab pegol resulted in improvements in uveitis and systemic symptoms. Moreover, residual chalazia and blepharitis also improved.

## Conclusions

We reported a rare case of unusually large, bilateral, multiple, and treatment-resistant chalazia that developed during adalimumab therapy for uveitis associated with GPP. While chalazia have not been previously reported as an adverse effect of adalimumab, such atypical presentations may suggest underlying immune dysregulation. In this case, the chalazia improved following a switch to certolizumab pegol due to secondary failure of adalimumab, further supporting a possible role of adalimumab-induced immune imbalance in the development of chalazia. This case underscores the importance of considering immune dysregulation as a potential underlying mechanism in atypical chalazia arising during biologic therapy.
